# Combination therapies with thiazolidinediones are associated with a lower risk of acute exacerbations in new-onset COPD patients with advanced diabetic mellitus: a cohort-based case–control study

**DOI:** 10.1186/s12890-021-01505-7

**Published:** 2021-04-29

**Authors:** Kuan-Yuan Chen, Sheng-Ming Wu, Chien-Hua Tseng, Kang-Yun Lee, Yu-Huei Lin, Hung-Yi Liu, Li-Nien Chien

**Affiliations:** 1 Graduate Institute of Clinical Medicine, College of Medicine, Taipei Medical University, Taipei, Taiwan; 2 Division of Pulmonary Medicine, Department of Internal Medicine, Shuang Ho Hospital, Taipei Medical University, New Taipei City, Taiwan; 3 Division of Pulmonary Medicine, Department of Internal Medicine, School of Medicine, College of Medicine, Taipei Medical University, Taipei, Taiwan; 4 Institute of Epidemiology and Preventive Medicine, College of Public Health, National Taiwan University, Taipei, Taiwan; 5 Post-Baccalaureate Program in Nursing, College of Nursing, Taipei Medical University, Taipei, Taiwan; 6 Office of Data, Taipei Medical University, No. 250 Wuxing St., Taipei, 11031 Taiwan; 7 School of Health Care Administration, College of Management, Taipei Medical University, Taipei, Taiwan

**Keywords:** Oral antihyperglycaemic drugs **(**OADs), Thiazolidinediones (TZDs), Type 2 diabetes mellitus (T2DM), Acute exacerbations of chronic obstructive pulmonary disease (AECOPD)

## Abstract

**Background:**

The effects of oral antihyperglycaemic drugs (OADs) for type 2 diabetes mellitus (T2DM) on the outcomes of co-existing chronic obstructive pulmonary disease (COPD) patients are not well studied. We examined the association of combinational OADs and the risk of acute exacerbations of COPD (AECOPD) in T2DM patients with co-existing COPD.

**Methods:**

A cohort-based case–control study was conducted using data from the National Health Insurance Research Database of Taiwan. Among new-onset COPD-T2DM patients, 65,370 were prescribed metformin and 2nd-line OADs before the date of COPD onset. Each AECOPD case was matched to 4 randomly selected controls according to the propensity score estimated by the patient’s baseline characteristics. Conditional logistic regression analysis was performed to estimate the association between AECOPD risk and OAD use.

**Results:**

Among COPD-T2DM patients, 3355 AECOPD cases and 13,420 matched controls were selected. Of the patients treated with a double combination of oral OADs (n = 12,916), those treated with sulfonylurea (SU) and thiazolidinediones (TZD) had a lower AECOPD risk than the patients who received metformin (MET) and SU, with an adjusted odds ratio (OR) of 0.69 (95% confidence interval [CI] 0.51–0.94, *P* = 0.02). Of the patients with a triple combination of oral OADs (n = 3859), we found that those treated with MET, SU and TZD had a lower risk of AECOPD (adjusted OR 0.81 (0.68–0.96, *P* = 0.01) than a combination of MET, SU and α-glucosidase inhibitors (AGIs) regardless of the level of COPD complexity.

**Conclusion:**

Combination therapies with TZD were associated with a reduced risk of AECOPD in advanced T2DM patients with co-existing COPD.

## Background

Multiple comorbidities of type 2 diabetes (T2DM) are common, and only 14% of patients may have no other comorbidities [1]. When considering the impact of different classes of drugs on comorbidities, the complexity of choosing therapeutic drugs for glycaemic control increases. Chronic obstructive pulmonary disease (COPD) is a common comorbidity in patients with T2DM, and approximately 10% of T2DM patients have COPD [2, 3]. Recent studies have shown that pre-existing diabetes or incident diabetes may worsen the risk of death in patients with COPD or acute exacerbations of COPD (AECOPD) [4, 5]. Therefore, optimizing DM care in the COPD population is imperative.

The recently updated guideline from the American Diabetes Association (ADA) recommends metformin, if not contraindicated and if tolerated, as the preferred initial oral antihyperglycaemic drug (OAD) for the treatment of T2DM [6]. As the progressive natural course of T2DM, when metformin monotherapy is no longer effective, the majority of advanced T2DM patients require a combination of different 2nd OADs or insulin therapy to achieve and maintain optimal glycaemic control. The ADA does not prioritize specific 2nd drugs based on their efficacy, side effects and impact on comorbidities except for cardiovascular and renal effects [6].

Whether strict blood glucose control in COPD-T2DM patients can improve the outcomes of COPD is unclear, but poor sugar control worsens the severity and clinical course of COPD based on previous studies [7, 8]. Although previous studies have demonstrated the impact of OADs on the clinical outcomes of COPD, such as metformin (MET) and thiazolidinediones (TZDs) [9–11], relatively few studies have focused on the effect of glucose-lowering agents on COPD outcomes in T2DM patients, particularly in patients with poor glycaemic control requiring add-on therapy to MET. At present, a knowledge gap remains in choosing the best drugs that are conducive to glycaemic control and can improve the clinical efficacy of new-onset COPD in patients with advanced T2DM. Therefore, the aim of this study was to examine the impact of add-on OADs on AECOPD risk in new-onset COPD patients with advanced T2DM who required combinational therapy. We conducted a cohort-based case–control study using data from the National Health Insurance Research Database (NHIRD) of Taiwan.

## Methods

### Data source

The NHIRD is a nationwide claim-based database of the National Health Insurance (NHI) programme provided by the National Health Insurance Administration (NHIA) of Taiwan. The NHI programme was launched in 1995 and is a compulsory insurance programme that provides reimbursement for most medical services and more than 30,000 prescription drugs. The data used in this study were collected between 2000 and 2015 and were maintained by the Health and Welfare Data Science Center (HWDC), Ministry of Health and Welfare, Executive Yuan, Taiwan. The NHIRD database includes information on inpatient, outpatient and drug prescription claims and uses the International Classification of Diseases, Ninth Revision, Clinical Modification (ICD-9-CM) and the Anatomical Therapeutic Chemical (ATC) system to define whether the patients had a specific disease diagnosis or drug prescription. To validate the accuracy of the diagnosis and the rationality of the treatment, the NHIA also routinely took samples and reviewed some of the NHI claims. Moreover, hospitals and clinics are penalized if patients receive unnecessary treatment. Each patient also has a unique encrypted identifier linked to the National Death Registry under the provisions of HWDC. This study was approved by the Joint Institutional Review Board of Taipei Medical University (approval no. N201808075).

### Study cohort

The initial cohort included new-onset COPD patients with diabetes between 2003 and 2014. If a patient had at least three disease diagnosis requirements within one year of follow-up, a washout period of at least three years was used to ensure that the patient was newly diagnosed with COPD. Then, we excluded patients (1) whose sex was unknown, who were not Taiwanese citizens or who were younger than 40 years old; (2) had no COPD prescription requirements within one year after the first diagnosis of COPD; (3) had a disease history of malignant tumour, asthma, chronic kidney disease, and renal dialysis; and (4) were diagnosed with type 1 diabetes before the first COPD diagnosis or no antidiabetic prescription statement or received MET monotherapy and received insulin therapy. The subsequent exclusion was to increase the homogeneity of the study population.

### Case and control patient selection

A general consensus on the definition of AECOPD is lacking. Generally, the definition of AECOPD is based on increased symptoms and/or increased utilization of health care. Based on previous studies [12, 13], we used the following approach in this claim-based study to identify patients with AECOPD as those that (1) had a hospital admission or an emergency visit due to COPD and required oral or injection corticosteroid (CS) or (2) received oral or injection CS therapy at a new visit. To increase the comparability, matched controls were selected based on incidence density sampling, which involved matching each AECOPD case with a sample of those potential controls at the time of case occurrence. Before matching, we additionally excluded patients who received monotherapy and then included subjects with double or triple combination OAD therapy whose regimen has been validated by clinical trials and meta-analyses [14]. Finally, each case was matched to 4 randomly selected controls according to the propensity score estimation by sex, age, year of COPD diagnosis, initial year of DM status, previous and coexisting disease conditions, Charlson comorbidity index (CCI), level complexity of COPD and COPD medication use three months prior to the date of AECOPD. The initial year of DM status was defined based on the first claim year of the patient initially receiving 2nd-line OADs continuously for at least 3 months. Because AECOPD did not occur in the control patients, we randomly assigned the surrogate event dates, which corresponded to the index date of their matched cases. We used this method to create a basis for the comparison of OAD exposure between the case and control patients.

### Exposure to oral antihyperglycaemic drugs (OADs)

We examined all OAD prescription records within three months before the index date of AECOPD in cases and pseudo-AECOPD in controls. We investigated the types of OAD, including MET, sulfonylurea (SU), α-glucosidase inhibitors (AGIs), TZDs and dipeptidyl peptidase-4 inhibitors (DPP-4i). The aim of our study was to determine the best drug as an add-on OAD to monotherapy for progressive T2DM in the context of considering the impact on the outcome of COPD. Then, we further categorized T2DM-COPD patients using a double or triple combination of OADs.

### Potential confounding variables

Previous or coexisting medical conditions were recorded if patients were diagnosed with chronic artery disease (CAD), hypertension (HTN), congestive heart failure (CHF), atrial fibrillation (AF), pneumonia, chronic liver disease (CLD), dementia/Parkinson’s disease and osteoporosis. Additionally, CCI is also considered a major risk, which represents the severity of comorbid conditions. The CCI in this study was modified because all patients were diagnosed with diabetes and COPD but had no history of malignancy. According to a previous study [15], we categorized the patients into low, moderate and high complexity by adjusting the severity of COPD and further divided the patients into low and moderate/high complexity groups due to a small sample size of high complexity. In addition, we also considered the history of COPD medication use in AECOPD cases and non-AECOPD controls [15], including short-acting beta agonists (SABAs), short-acting muscarinic antagonists (SAMAs), long-acting beta agonists (LABAs), long-acting muscarinic antagonists (LAMAs) and inhaled corticosteroids (ICSs).

### Statistical analysis

The baseline differences between case and control patients were measured by standardized mean difference (SMD) as previously described [16]. Conditional logistic regression was used to estimate the odds ratios (ORs), adjusted odds ratios (aORs), and 95% confidence intervals (CIs) for the association of AECOPD risk and OAD treatment. The statistical analyses were performed using SAS/STAT, Version 9.4, (SAS Institute, Cary, NC, USA) and STATA 13 (Stata Corp, College Station, TX, USA). A *P* value < 0.05 and SMD > 0.1 were set as the level of statistical significance.

## Results

### Baseline characteristics

**Fig. 1:**
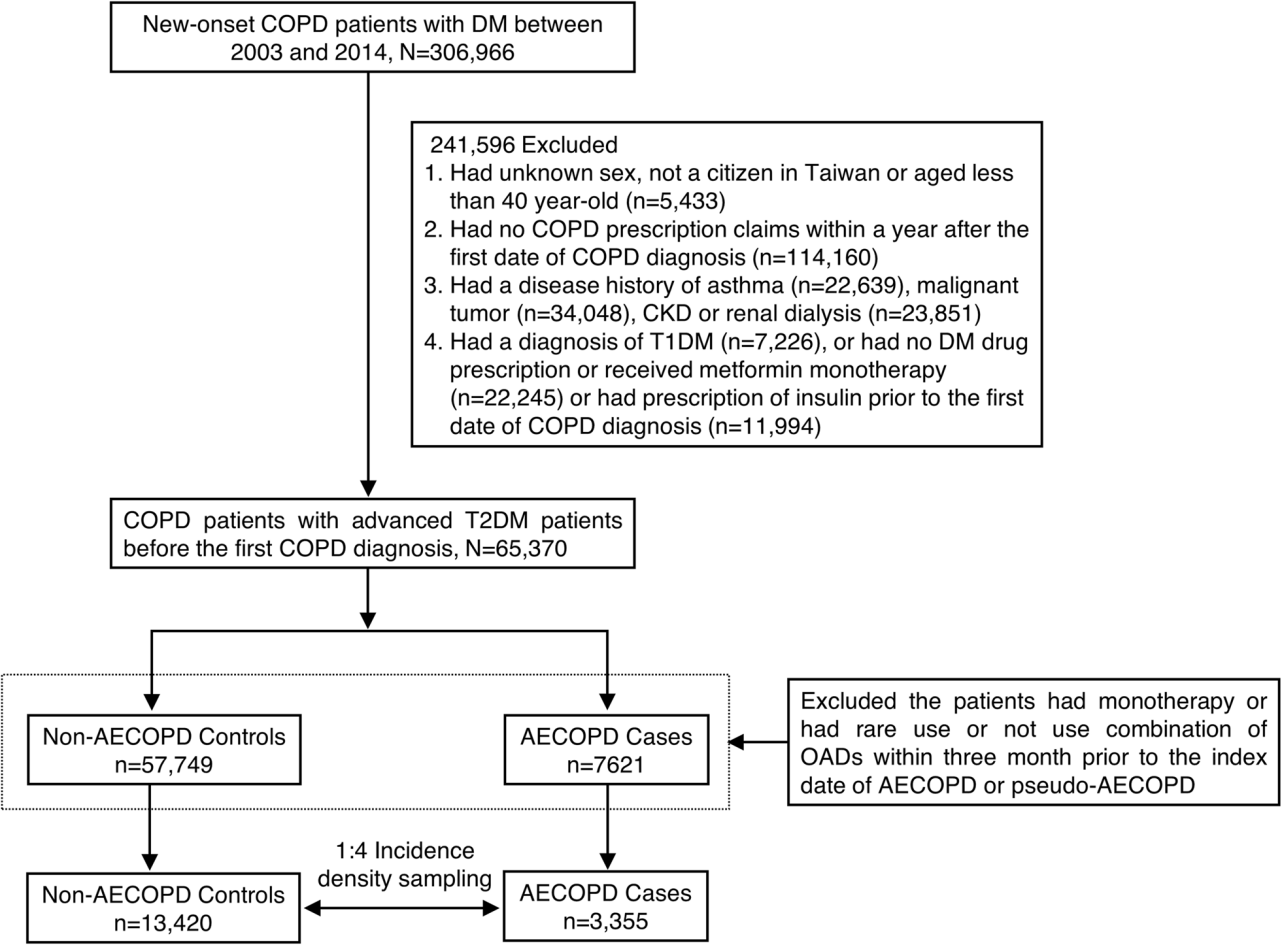
Patient selection process. *AECOPD* acute of exacerbations chronic obstructive pulmonary disease, *CKD* chronic kidney disease, *COPD* chronic obstructive pulmonary disease, *DM* diabetes mellitus, *OADs* oral antihyperglycemic drugs, *T1DM* type 1 diabetes mellitus, *T2DM* type 2 diabetes mellitus

Of new-onset COPD patients with advanced T2DM, 3355 AECOPD cases and 13,420 non-AECOPD matched controls were selected using an incidence density sampling method (Fig. 1). The baseline characteristics of the case and control patients are shown in Table 1. In the COPD-T2DM cohort, two-thirds of AECOPD cases were male, and the mean age was 72 years old (SD: 10.5). The 3 most common previous or coexisting disease conditions were HTN (65.0%), CAD (20.8%) and pneumonia (12.9%), and 42.1% had a modified CCI between 1 and 2. In terms of the level of COPD complexity at initial diagnosis, the AECOPD patients were divided into low-level (51.3%) and moderate/high-level (48.7%) groups and the non-AECOPD controls were classified into low-level (49.8%) and medium/high-level groups (50.2%). For COPD medication three months before the index date of AECOPD, the majority of patients had received ICS or steroids, and only 3% of the patients received either SABA, SAMA, LABA or LAMA. Because we used the propensity score approach to adjust the baseline characteristics of AECOPD cases and non-AECOPD controls, the groups did not differ in the variables listed in Table 1.

**Table 1:** Baseline difference between AECOPD case patients and non-AECOPD control T2DM patients

Variables	Non-AECOPD controls		AECOPD cases		SMD
	n	(%)	n	(%)	
Sample size	13,420		3355		
Male, yes	8956	(66.7)	2239	(66.7)	0.000
*Age, [mean, SD]*	[72.2, 10.4]		[72.1, 10.5]		
40–49	350	(2.6)	92	(2.7)	0.008
50–59	1437	(10.7)	357	(10.6)	0.002
60–69	2902	(21.6)	759	(22.6)	0.024
70–79	5318	(39.6)	1266	(37.7)	0.039
≥ 80	3413	(25.4)	881	(26.3)	0.019
*Year of the first DM claim*					
2000–2002	8059	(60.1)	2035	(60.7)	0.012
2003–2006	3114	(23.2)	769	(22.9)	0.007
2007–2010	1681	(12.5)	418	(12.5)	0.002
2011–2014	566	(4.2)	133	(4.0)	0.013
*Year of COPD diagnosis*					
2003–2006	4411	(32.9)	1121	(33.4)	0.012
2007–2010	4906	(36.6)	1228	(36.6)	0.001
2011–2014	4103	(30.6)	1006	(30.0)	0.013
*Previous or coexisting disease conditions, yes*					
HTN	8671	(64.6)	2182	(65.0)	0.009
CAD	2867	(21.4)	698	(20.8)	0.014
CHF	1109	(8.3)	282	(8.4)	0.005
AF	581	(4.3)	154	(4.6)	0.013
Pneumonia	1761	(13.1)	433	(12.9)	0.006
CLD	849	(6.3)	216	(6.4)	0.005
Dementia/Parkinson	1425	(10.6)	364	(10.8)	0.007
Osteoporosis	447	(3.3)	114	(3.4)	0.004
*CCI, [mean, SD]*	[1.5, 1.5]		[1.6, 1.5]		
0	4294	(32.0)	1078	(32.1)	0.003
1–2	5893	(43.9)	1412	(42.1)	0.037
≥ 3	3233	(24.1)	865	(25.8)	0.039
*Level of COPD complexity at initial diagnosis*					
Low	6689	(49.8)	1,722	(51.3)	0.030
Moderate/high	6731	(50.2)	1633	(48.7)	0.030
*COPD medication use 3 month prior to index date, yes*					
SABA or SAMA	169	(1.3)	39	(1.2)	0.009
LABA or LAMA	417	(3.1)	60	(1.8)	0.085
ICS or Steroid	12682	(94.5)	3210	(95.7)	0.054
Others	152	(1.1)	46	(1.4)	0.021

*AECOPD* acute exacerbations of chronic obstructive pulmonary disease, *AF *atrial fibrillation, *CAD* coronary artery disease, *CCI* Charlson comorbidity index, *CHF* congestive heart failure, *CLD* chronic liver disease, *COPD* chronic obstructive pulmonary disease, *DM* diabetes mellitus, *HTN* hypertension, *SMD* standardized mean difference, *SABA* short-acting beta agonists, *SAMA* short-acting muscarinic antagonist, *LABA* long-acting beta agonists, *LAMA* long-acting muscarinic antagonist, *ICS* Inhaled corticosteroids

^*^SMD = difference in means or proportions divided by standard error; imbalance defined as absolute value greater than 0.1

### OADs use and the risk of AECOPD

Among the COPD-T2DM patients treated with a combination of oral OADs (n = 12,916), compared to patients who had received both MET and SU, AECOPD patients were less likely to be treated with a combination of SU and TZD within three months before the date of AECOPD, with an adjusted odds ratio (OR) of 0.69 (95% confidence interval [CI] 0.51–0.94, *P* = 0.02) (Table 2). When further dividing the patients into low and moderate/high levels of COPD at the initial COPD diagnosis, we still found that a combination of SU and TZD was associated with a reduced risk of AECOPD in patients with lower COPD complexity (adjusted OR 0.20, 95% CI of 0.32–0.80).

**Table 2:** The association between double oral OADs use and risk of AECOPD in T2DM patients with COPD (n = 12,916)

Level of COPD complexity	Combination of OADs	Non-AECOPD controls		AECOPD cases		Odds ratio					
		n	(%)	n	(%)	Crude	(95% CI)	*P*	Adjusted*	(95% CI)	*P*
Overall											
	SU + AGI	526	(5.1)	111	(4.4)	0.87	(0.72–1.05)	0.15	0.87	(0.72–1.05)	0.14
	SU + DPP-4i	394	(3.8)	89	(3.5)	0.92	(0.74–1.14)	0.43	0.93	(0.75–1.15)	0.50
	SU + TZD	247	(2.4)	41	(1.6)	0.71	(0.52–0.97)	0.03	0.69	(0.51–0.94)	0.02
	MET + AGI	348	(3.4)	79	(3.1)	0.92	(0.74–1.16)	0.48	0.92	(0.73–1.15)	0.45
	MET + DPP-4i	816	(7.9)	190	(7.5)	0.94	(0.81–1.09)	0.43	0.95	(0.82–1.11)	0.51
	MET + TZD	238	(2.3)	60	(2.4)	1.00	(0.78–1.30)	0.98	1.01	(0.78–1.30)	0.96
	MET + SU	7816	(75.3)	1961	(77.5)	1.00	(Ref.)		1.00	(Ref.)	
Moderate/high											
	SU + AGI	290	(5.5)	49	(4.0)	0.75	(0.56–1.00)	0.05	0.77	(0.58–1.03)	0.08
	SU + DPP-4i	232	(4.4)	40	(3.3)	0.76	(0.55–1.04)	0.09	0.78	(0.57–1.07)	0.13
	SU + TZD	95	(1.8)	23	(1.9)	1.01	(0.67–1.53)	0.97	0.99	(0.66–1.51)	0.98
	MET + AGI	172	(3.3)	47	(3.8)	1.11	(0.83–1.49)	0.48	1.16	(0.86–1.56)	0.32
	MET + DPP-4i	375	(7.1)	78	(6.4)	0.89	(0.71–1.12)	0.33	0.89	(0.70–1.13)	0.34
	MET + TZD	92	(1.7)	26	(2.1)	1.14	(0.77–1.68)	0.51	1.15	(0.78–1.70)	0.48
	MET + SU	4004	(76.1)	959	(78.5)	1.00	(Ref.)		1.00	(Ref.)	
Low											
	SU + AGI	236	(4.6)	62	(4.7)	1.00	(0.77–1.29)	1.00	0.96	(0.74–1.24)	0.76
	SU + DPP-4i	162	(3.2)	49	(3.7)	1.12	(0.84–1.49)	0.45	1.11	(0.83–1.47)	0.49
	SU + TZD	152	(3.0)	18	(1.4)	0.51	(0.32–0.81)	< 0.01	0.50	(0.32–0.80)	< 0.01
	MET + AGI	176	(3.4)	32	(2.4)	0.74	(0.52–1.05)	0.09	0.74	(0.52–1.05)	0.10
	MET + DPP-4i	441	(8.6)	112	(8.6)	0.97	(0.80–1.18)	0.78	0.99	(0.81–1.21)	0.90
	MET + TZD	146	(2.8)	34	(2.6)	0.91	(0.64–1.28)	0.58	0.93	(0.66–1.32)	0.70
	MET + SU	3812	(74.4)	1002	(76.5)	1.00	(Ref.)		1.00	(Ref.)	

^*^Adjusted for age, sex, DM status, previous and coexisting disease conditions, modified CCI, complexity of COPD and COPD medications listed in Table 1

*AECOPD* acute exacerbations of chronic obstructive pulmonary disease, *AGI* α-glucosidase inhibitors, *CCI* Charlson comorbidity index, *CI* confidence interval, *DPP‐4i* dipeptidyl peptidase‐4 inhibitor, *DM* diabetes, *MET* metformin, *OADs* oral antihyperglycemic drugs, *OR* odd ratio, *Ref.* reference group, *SU* sulfonylurea, *TZD* thiazolidinediones

For the patients treated with a triple combination of OADs (n = 3859), we found that AECOPD patients were less likely to have been treated with MET, SU and TZD, compared to MET, SU and AGI, with an adjusted OR of 0.81 (95% CI 0.68–0.96, *P* = 0.01) (Table 3). Similar results were found at different levels of COPD complexity; however, the finding was significant for patients with a moderate/high level.

**Table 3:** The association between triple oral OADs use and risk of AECOPD in T2DM patients with COPD (n = 3859)

Level of COPD complexity	Combination of OADs	Non-AECOPD controls		AECOPD cases		Odds ratio					
		n	(%)	n	(%)	Crude	(95% CI)	*P*	Adjusted*	(95% CI)	*P*
Overall											
	MET + SU + TZD	1074	(35.4)	243	(29.5)	0.81	(0.68–0.95)	0.01	0.81	(0.68–0.96)	0.01
	MET + SU + DPP-4i	933	(30.7)	276	(33.5)	1.00	(0.85–1.17)	0.98	1.02	(0.87–1.21)	0.78
	MET + SU + AGI	1028	(33.9)	305	(37.0)	1.00	(Ref.)		1.00	(Ref.)	
Moderate/high											
	MET + SU + TZD	479	(32.6)	104	(25.3)	0.76	(0.60–0.97)	0.03	0.75	(0.58–0.96)	0.02
	MET + SU + DPP-4i	440	(29.9)	138	(33.6)	1.02	(0.81–1.28)	0.87	1.06	(0.84–1.34)	0.63
	MET + SU + AGI	552	(37.5)	169	(41.1)	1.00	(Ref.)		1.00	(Ref.)	
Low											
	MET + SU + TZD	595	(38.0)	139	(33.7)	0.85	(0.67–1.08)	0.18	0.88	(0.79–1.28)	0.28
	MET + SU + DPP-4i	493	(31.5)	138	(33.4)	0.98	(0.78–1.25)	0.89	1.00	(0.69–1.11)	0.97
	MET + SU + AGI	476	(30.4)	136	(32.9)	1.00	(Ref.)		1.00	(Ref.)	

^*^Adjusted for age, sex, DM status, previous and coexisting disease conditions, modified CCI, complexity of COPD and COPD medications listed in Table 1

*AECOPD* acute exacerbations of chronic obstructive pulmonary disease, *AGI* α-glucosidase inhibitors, *CCI* Charlson comorbidity index, *CI* confidence interval, *DPP‐4i* dipeptidyl peptidase‐4 inhibitor, *DM* diabetes, *MET* metformin, *OADs* oral antihyperglycemic drugs, *OR* odd ratio, *Ref.* reference group, *SU* sulfonylurea, *TZD* thiazolidinediones

## Discussion

In the present study, we showed that COPD-T2DM patients with OAD use were treated only for glycaemic sugar control, while a double combination of SU and TZDs and a triple combination of MET, SU, and TZDs were correlated with a decreased risk of AECOPD. The results were fairly consistent in patients with moderate or high complexity COPD.

In the COPD-T2DM cohorts, our analysis results revealed that add-on TZDs could decrease the risk of AECOPD in patients receiving double and triple OAD combinations. The results are consistent with previous findings that TZDs are associated with a reduced risk of AECOPD after adjusting for the severity of DM itself, which may have a significant effect on AECOPD [11]. Here, we further considered the effect of COPD severity on AECOPD using COPD complexity classification. To rule out the effect of COPD medications on AECOPD, we found that TZD has a similar effect on reducing the frequency of AECOPD after adjusting for confounding factors.

COPD-T2DM is considered a syndrome that can share risk factors (such as smoking) [17], genes (such as β_2_-adrenergic receptor gene, ADRB2) [18], proteins (such as Nod-like receptor containing a pyrin domain 3, NLRP3) [19, 20] and pathways (such as systemic inflammation and oxidative stress) [21–23]. Although the underlying mechanism of these shared components is complex and has not been fully elucidated, the important common approach for concurrently treating COPD and T2DM to target systemic inflammation would be a reasonable therapeutic strategy [24].

In addition to the function of lowering glycaemic sugar, some OADs may also have anti-inflammatory activity due to their pleiotropic effects [25, 26]. TZD is an OAD with anti-inflammatory activity. Since the late 1990s, TZD has been studied and has been used in combination with MET to treat T2DM [27]. The anti-inflammatory effects of TZD occur through cellular mechanisms that activate the nuclear transcription factor peroxisome proliferator-activated receptor gamma (PPAR-γ) and, at least in part, glucocorticoid nuclear translocation [28, 29]. AECOPD patients with frequent exacerbations have more inflamed existing airways and systemic inflammation and poor inflammation resolution [30–34]. Therefore, TZDs may exert anti-inflammatory effects that prevent the pro-inflammatory status of AECOPD. Additionally, the major comorbidities of COPD-T2DM, such as CVD, may induce or worsen AECOPD [35]. TZDs also exert important functions in regulating vascular inflammation through PPAR-γ activation and inhibit vascular smooth muscle proliferation, thereby having an effect against atherosclerosis [36–38]. The protective role of TZDs on cardiovascular outcomes may contribute to reducing CVD-related AECOPD, particularly pioglitazone [39, 40].

Previous studies have shown that MET use can reduce the utilization of health care in COPD-T2DM patients and reduce its adverse prognostic effects [9, 10]; however, no significant beneficial effect of combination therapy with MET use was found in our research. The major causes of the different results can be attributed to the enrolled and analysed patients. The favourable effect of MET on reducing COPD-specific health care utilization was only presented in COPD patients with lower complexity but not moderate to high complexities, and the study did not show the effect of mono- or combinational therapy with MET. In our study, we clearly defined patients treated with combination therapy including, MET and the data demonstrated no effect on reducing the risk of AECOPD in all COPD complexities. COPD-T2DM patients may have better survival outcomes with MET treatment; however, the consequence may be confounded by COPD severity and the medication regimen for COPD.

DPP-4 is another target of OAD that can drive the T helper type 1 (Th1) immune response and is also considered to be involved in COPD pathogenesis [41]. Additionally, the active protease DDP-4/CD26 may act on CXCL12, which is associated with exacerbating tissue damage in COPD [42]. Therefore, COPD may be better controlled using an inhibitor of DDP-4 (DDP-4i); however, our results did not show significant differences in the risk of AECOPD when therapy was combined with DDP-4i.

Our study suggests that TZDs are a better choice for combinational therapy when glycaemic control deteriorates from initial control in COPD-T2DM patients. This recommendation was based on a more strictly defined patient population, controlled for important clinical confounders and considering the impact of OADs on AECOPD. Our study does have some limitations. The use of ICS in COPD plays an important role in glycaemic level control. No statistically significant change in the HbA1c level was found in a small prospective randomized, double-blind placebo-control, 42-day short-term study [43]; however, a large retrospective study showed that long-term use of ICS for the treatment of COPD or asthma was correlated with the progression of diabetes [44]. In addition, the effect of ICS on the deterioration of glycaemic control may be related to the dose [45]. The efficacy and safety between the two more frequently used ICSs (fluticasone and budesonide) have been reported; but, only fluticasone showed a dose-related increase in the risk of pneumonia in COPD and may induce stress hyperglycaemia [46]. In general, ICS is usually prescribed to patients with more severe and frequent exacerbations of COPD, and systemic inflammation might also be related to elevated glycaemic levels [21]. The role of ICS and specifically the different types of ICS used on AECOPD in the different classes of OADs is important to evaluate. Unfortunately, in the current study, we only analysed ICS use as a confounding factor when assessing the association between TZD and AECOPD. Thus, the assessment of ICS remains to be further investigated. Second, data on drug exposure were obtained from prescription records, which may not reflect actual usage. According to the 2017 GOLD guidelines, a fixed combination of LAMA/LABA is recommended as the first-line treatment for COPD, and ICS is recommended as an additional treatment under specific conditions [47]. Moreover, ICS is considered to be the first-line treatment for COPD patients with severe airflow limitation [48]. In this study, there was a low use of bronchodilators and a high use of ICS alone in COPD from 2003 to 2014, which may be because the dual bronchodilator was first approved in Taiwan in 2014. ICS alone may not truly be used alone because different combinational inhaler therapies were difficult to recognize from our database. Moreover, the trend was consistent with the higher use of ICS/LABA treatment for COPD in real-world data before the change in the GOLD guidelines in 2017 [49]. Third, the NHIRD database lacks other important clinical information, such as smoking, certain vaccinations, and medication compliance, which may lead to AECOPD occurrence. Fourth, the administrative claims database from which the NHIRD sample was derived did not consider certain clinical characteristics, such as the severity of COPD. Thus, we chose new-onset COPD patients to decrease the bias due to COPD severity. In addition, we applied the cross-sectional analysis developed by Mapel et al. [15] to adjust the potential effect of the level of COPD severity on AECOPD risk. We recognized that some uncontrolled influences remain that may affect the results of our study. Finally, this study included a cohort of Taiwanese patients and therefore may not be generalizable to other populations due to variations in genetics and treatment guidelines for both diseases in other areas. Future prospective studies on the effects of TZDs are warranted to confirm our findings.

## Conclusion

These results showed that combination therapy with TZDs is associated with a reduced risk of AECOPD regardless of double or triple combinational regimens in COPD-T2DM patients, particularly in moderate to severe complexity COPD populations. The number of T2DM patients with co-existing COPD may increase in the future. TZDs play different protective roles for both diseases and are suggested to be used in these patients, but prospective randomized controlled trials are needed to verify our results.

## Data Availability

The dataset used and/or analyzed during the current study are available from the corresponding author on reasonable request.

## Abbreviations

COPD: Chronic obstructive pulmonary disease
OADs: Oral antihyperglycemic drugs
TZDs: Thiazolidinediones
CCI: Charlson comorbidity index
DPP-4i: Dipeptidyl peptidase-4 inhibitor
T2DM: Type 2 diabetes mellitus
AECOPD: Acute exacerbations of chronic obstructive pulmonary disease
MET: Metformin
SU: Sulfonylurea
OR: Odds ratio
aOR: Adjusted odds ratios
CI: Confidence interval
AGI: α-Glucosidase inhibitors
CAD: Chronic artery disease
HTN: Hypertension
CHF: Congestive heart failure
CLD: Chronic liver disease
SABA: Short-acting beta-agonists
SAMA: Short-acting muscarinic antagonists
LABAs: Long-acting beta-agonists
LAMAs: Long-acting muscarinic antagonists
ICS: Inhaled corticosteroids
NLRP3: Nod-like receptor containing a pyrin domain
PPAR-γ: Peroxisome proliferator-activated receptor gamma
Th1: T helper type 1
CXCL12: C-X-C motif chemokine ligand 12
